# Animal Metabolite Database: Metabolite Concentrations in Animal Tissues and Convenient Comparison of Quantitative Metabolomic Data

**DOI:** 10.3390/metabo13101088

**Published:** 2023-10-17

**Authors:** Vadim V. Yanshole, Arsenty D. Melnikov, Lyudmila V. Yanshole, Ekaterina A. Zelentsova, Olga A. Snytnikova, Nataliya A. Osik, Maxim V. Fomenko, Ekaterina D. Savina, Anastasia V. Kalinina, Kirill A. Sharshov, Nikita A. Dubovitskiy, Mikhail S. Kobtsev, Anatolii A. Zaikovskii, Sofia S. Mariasina, Yuri P. Tsentalovich

**Affiliations:** 1 Laboratory of Proteomics and Metabolomics, International Tomography Center SB RAS, Institutskaya Str. 3a, Novosibirsk 630090, Russia; melnikov.arsenty@tomo.nsc.ru (A.D.M.); lucy@tomo.nsc.ru (L.V.Y.); zelentsova@tomo.nsc.ru (E.A.Z.); koa@tomo.nsc.ru (O.A.S.); n.osik@tomo.nsc.ru (N.A.O.); m.fomenko@tomo.nsc.ru (M.V.F.); kate.sormacheva@tomo.nsc.ru (E.D.S.); a.kalinina@tomo.nsc.ru (A.V.K.); yura@tomo.nsc.ru (Y.P.T.); 2 Department of Physics, Novosibirsk State University, Pirogova Str. 1, Novosibirsk 630090, Russia; 3 Laboratory of Molecular Epidemiology and Biodiversity of Viruses, Federal Research Center of Fundamental and Translational Medicine, Timakova Str. 2, Novosibirsk 630117, Russia; sharshov@yandex.ru (K.A.S.); nikitadubovitskiy@frcftm.ru (N.A.D.); 4 Department of Information Technologies, Novosibirsk State University, Pirogova Str. 1, Novosibirsk 630090, Russia; m.kobtsev@g.nsu.ru; 5 Department of Mathematics and Computer Science, Saint Petersburg State University, 14th Line V. O. 29, Saint Petersburg 199178, Russia; anat097@mail.ru; 6 Department of Chemistry, Lomonosov Moscow State University, Moscow 119991, Russia; sofia.mariasina@yandex.ru; 7 Faculty of Fundamental Medicine, Lomonosov Moscow State University, Moscow 119991, Russia; 8 RUDN University, Miklukho-Maklaya Str. 6, Moscow 117198, Russia

**Keywords:** database, quantitative metabolomics, data reuse, data exchange, animal tissues, NMR spectroscopy, LC-MS

## Abstract

The Animal Metabolite Database (AMDB, https://amdb.online) is a freely accessible database with built-in statistical analysis tools, allowing one to browse and compare quantitative metabolomics data and raw NMR and MS data, as well as sample metadata, with a focus on the metabolite concentrations rather than on the raw data itself. AMDB also functions as a platform for the metabolomics community, providing convenient deposition and exchange of quantitative metabolomic data. To date, the majority of the data in AMDB relate to the metabolite content of the eye lens and blood of vertebrates, primarily wild species from Siberia, Russia and laboratory rodents. However, data on other tissues (muscle, heart, liver, brain, and more) are also present, and the list of species and tissues is constantly growing. Typically, every sample in AMDB contains concentrations of 60–90 of the most abundant metabolites, provided in nanomoles per gram of wet tissue weight (nmol/g). We believe that AMDB will become a widely used tool in the community, as typical metabolite baseline concentrations in tissues of animal models will aid in a wide variety of fundamental and applied scientific fields, including, but not limited to, animal modeling of human diseases, assessment of medical formulations, and evolutionary and environmental studies.

## 1. Introduction

The combination of the genome, transcriptome, proteome, and metabolome determines the phenotype of any species [1]. Metabolomics is one of the youngest fields of ‘-omics’ sciences, aimed at identifying and quantifying as many metabolites as possible in biological tissues and fluids. The metabolome is very susceptible to many external and internal factors for an individual as compared to the genome, transcriptome, or proteome; for that reason, metabolomics is used in a wide range of fundamental and applied scientific fields. In particular, the metabolomic approach turns out to be extremely useful for medical applications, including diagnosis, verification of the efficiency of drug administration, and understanding the mechanisms of the development of a wide variety of diseases [2,3,4,5,6]. The existing metabolomic databases are mainly related to human tissues and fluids. For example, the huge Human Metabolome Database, HMDB [7] contains data on over 250,000 metabolites found or predicted to be present in the human body. These data include not only the presence and concentrations of metabolites in human tissues but also their NMR and MS spectra, physical properties, participation in biochemical pathways, and other data collected from thousands of books, articles, and databases.

Unfortunately, metabolomic data on animal tissues other than humans are much scarcer. The exceptions are laboratory animals (mice, rats, rabbits): metabolomic analysis of their biological fluids and tissues is often used for studying the metabolic response to different factors (genetic differences, diseases, drug administration, environmental changes, and so on [8]). A significant part of these studies is performed using LC-MS or GC-MS methods, which usually yield semi-quantitative results, i.e., the content of metabolites in the experimental groups relative to the control groups. One can find quantitative metabolomic data (absolute values of metabolite concentrations in a tissue) relatively rarely, even for laboratory animals. For wild animals, the situation is much worse: in modern literature, quantitative data are published for tissues of very few species. One should note that quantitative metabolomic data are significantly more valuable than semi-quantitative ones since only quantitative data are available for future ‘eternal’ reuse, the addition of new samples or groups, the realization of new comparisons, data mining, new interpretations, and more [9]. Quantitative data can be obtained with the use of NMR spectroscopy, which suffers from relatively low sensitivity, but the peak areas of the metabolite signals in NMR spectra are directly proportional to the compound concentrations [10,11]. That makes the determination of the metabolite content in tissues rather easy and straightforward with the use of only one internal standard. The use of high magnetic fields and cryogenic probes can significantly improve the sensitivity of the NMR method. The more sensitive LC/GC-MS method also allows for obtaining quantitative data, but this requires the construction of calibration curves or the use of isotopically-labeled standards for every metabolite under study, which is rather labor-consuming.

Nowadays, the scientific community and publishers often demand that raw data be publicly accessible. There are several repositories that allow the deposition and sharing of data from metabolomic experiments, the most popular being the MetaboLights (https://www.ebi.ac.uk/metabolights/, accessed on 7 October 2023, [12]) and the Metabolomics Workbench (https://www.metabolomicsworkbench.org/, accessed on 7 October 2023). However, a significant amount of deposited data is semi-quantitative and thus cannot be reused in a simple way [13,14,15]. In cases where the deposited data are quantitative, they often either contain a limited number of metabolites, or the raw data are deposited with a focus on the raw data itself without detailed tables describing the obtained results or concentrations for each identified and quantified metabolite. For that reason, the published raw data are not as useful for other researchers as they could be [16,17]. Moreover, different labs, and sometimes even researchers within the same lab, tend to keep their data in spreadsheets with special formats that are convenient only for them, making it harder to compare data with each other [16,18]. One more problem is that existing metabolomic data on different animal species are scattered across scientific literature and narrow databases [19,20,21,22,23,24], and finding data on a specific metabolite or a group of metabolites in a specific tissue of a specific species might be difficult. Therefore, there is an urgent need to create a publicly available database on the quantitative content of metabolites in tissues of animals from various taxonomic groups.

Such a database should also aid in solving issues concerning the modeling of human diseases in animals. Model animals are most often used when human tissues and fluids used as controls in metabolomic experiments are hard- or even impossible-to-reach (such as cerebrospinal fluid, brain, inner organs, etc.) due to ethical restrictions. The selection of the correct model animal for a disease is often challenging [8,25,26,27], and thus an inventory of baseline metabolite concentrations in the tissues of animals would be of great aid.

In this work, we present the Animal Metabolite Database (AMDB, https://amdb.online), a freely accessible database on the quantitative content of metabolites in the tissues of animals. The AMDB is designed to ease browsing and comparing quantitative metabolomics data and raw NMR and MS data, as well as sample metadata. Moreover, the AMDB is a platform serving the metabolomics community for the convenient deposition and exchange of ‘eternal’ quantitative metabolomics data.

To date, the AMDB generally covers the metabolite content of the tissues of vertebrates, primarily wild species from Siberia, Russia and laboratory rodents, with a focus on the eye lens and blood, although data on other tissues (muscle, heart, liver, brain, etc.) are also present. The list of species and tissues studied by our group is constantly growing. The concentrations of the most abundant metabolites (typically, 60–90 compounds for each sample) in tissues are obtained mostly with the NMR method and are given in nanomoles per gram of wet tissue weight (nmol/g).

We hope that groups around the world will join the use of the AMDB database and platform by sharing their own data, which will lead to more comprehensive data coverage and increase the data utility, and the AMDB will become a widely used tool in the metabolomics community. We believe that publicly available structured information on the concentrations of metabolites in tissues of various species, instant human-readable data overview, and corresponding raw data and metadata present in the AMDB would help scientists from a wide range of scientific fields and will open up opportunities for bioinformaticians to conduct large-scale experiments with deposited data.

## 2. Database Description and Content

### 2.1. Database Overview

The Animal Metabolite Database (AMDB) is a freely accessible database with built-in statistical analysis tools, and human-readable data visualization, providing information on the concentrations of metabolites in tissues and fluids of various animal species. Metabolite absolute quantitative content is provided in nanomoles per gram of wet tissue weight (nmol/g). To date, it primarily covers the metabolite content of the eye lens of vertebrates, although the list of species and tissues is constantly growing. A list of investigated species is available in the Species section of the website.

The core of the AMDB are the samples, which represent the main structural elements within the database. Each sample corresponds to a specific tissue from an individual animal and contains valuable information, first of all, the concentrations of the most abundant metabolites, and the raw data obtained from instruments (NMR or LC/GC-MS). Additionally, each sample includes valuable metadata encompassing species taxonomy, age, weight, sampling location, diseases, as well as various other examined factors, providing comprehensive information for analysis and interpretation. To enhance organization, samples that share similarities, such as belonging to the same species, or tissue type, and being associated with a particular disease or factor, are grouped together into sample groups. These sample groups consist of samples that exhibit a rather high degree of uniformity. The resulting sample groups are compared and analyzed in metabolomics experiments, allowing for a more comprehensive understanding of the data.

The AMDB website has the following sections **Species**, **Experiments**, **Groups**, **Samples**, **Metabolites**, **Metadata**, and **About**. Descriptions of these sections are provided below.

Currently, the AMDB contains data on 46 species, 14 tissues, 776 samples, 155 quantified metabolites, and more than 44,000 measured concentration values.

### 2.2. Obtaining Samples and Data for the AMDB

An important part of creating the AMDB is obtaining biological tissue samples from domestic, wild, and laboratory animals. To achieve this, our lab has established collaborations with several scientific labs, farms, and hunting enterprises throughout Siberia, the Far East, and several other regions of Russia. Wild species were primarily obtained from relatively clean areas, making the data valuable for ecological research. Tissue sampling in the field, laboratory, or farm was performed either with the participation of members of our team or according to guidelines provided by our group. These guidelines included strict adherence to the European Union Directive 2010/63/EU on the protection of animals used for scientific purposes, the Declaration of Helsinki—ethical principles for medical research involving human subjects, and requirements for metabolomic-oriented studies: tissue samples should be extracted from the body as soon as possible after the animal’s death, placed in a clean vial, frozen in liquid nitrogen, and stored at −70 °C until analyzed.

Sample preparation, methodology development, and verification are described in our previous papers [26,28,29,30]. Briefly, each sample was weighed to obtain 50–200 mg of wet tissue weight. The tissues were homogenized in glass vials using a TissueRuptor II rotor-stator homogenizer (Qiagen, Venlo, The Netherlands) with 1600 µL of cold MeOH (−20 °C), followed by the addition of 800 µL of water and 1600 µL of cold chloroform. The mixture was shaken for 20 min in a shaker and then left at −20 °C for 30 min. After that, the mixture was centrifuged at 16,100× *g*, +4 °C for 30 min, resulting in two immiscible liquid layers separated by a lipid-protein layer. The upper aqueous layer (MeOH-H_2_O) was collected, and divided into two parts—for NMR (2/3) and LC-MS (1/3) analyses, and vacuum-dried for further analysis.

For NMR measurements, the extracts were dissolved in 600 μL of D_2_O containing 2 × 10^–5^ M of sodium 4,4-dimethyl-4-silapentane-1-sulfonic acid (DSS) as an internal standard and 20 mM of deuterated phosphate buffer (pH 7.2). The ^1^H NMR measurements were performed using an AVANCE III HD 700 MHz NMR spectrometer (Bruker BioSpin, Rheinstetten, Germany). The NMR spectra for each sample in a standard 5 mm glass NMR tube were acquired using a 5 mm TXI ATMA NMR probe by summing 64–96 transients while maintaining the sample temperature at 25 °C and using a 90-degree detection pulse. To allow for the relaxation of all spins, a repetition time of 20 s was used between scans. Prior to the acquisition, low-power radiation was applied at the water resonance frequency to presaturate the water signal.

For LC-MS measurements, the extracts were dissolved in 150 μL of a solvent with as little influence on the starting conditions of LC runs as possible. For reversed-phase LC (C18), the solvent was the aqueous solution of 10 mM ammonium formate with the addition of 0.1% formic acid. In the case of hydrophilic interaction liquid chromatography (HILIC), the solvent was >50% acetonitrile in ammonium formate buffer. The LC separation was performed on an UltiMate 3000RS chromatograph (Dionex, Germering, Germany), equipped with a diode array UV-vis detector (DAD) with a 190–800 nm spectral range, and was used with a flow cell. After the DAD cell, the flow is guided either through the homemade splitter (1:10, if flow rate > 500 μL/min), or directly to one of the ESI-Q-TOF high-resolution hybrid mass spectrometers, either maXis 4G or Impact II (Bruker Daltonics, Bremen, Germany).

## 3. Browsing and Searching the AMDB

### 3.1. Species

The AMDB website features a dedicated section that allows users to browse or search for species or taxa using the loose taxonomy tree or the search bar. Taxa and species have detailed cards, containing descriptions, pictures, taxon dependencies, as well as lists of samples, groups, and experiments related to the taxon of interest. Additionally, species cards include statistical charts generated from all samples associated with a particular species throughout the database. Users can gather all the information for a single tissue of one or several species of interest and directly compare them within the AMDB based on the available database information (refer to Section 3.4.).

### 3.2. Samples, Groups, and Experiments

Each sample is associated with a unique card that provides a list of quantified metabolites and their concentration values. The sample card also includes metadata such as descriptions, species, tissue, information associated with diseases or factors, hyperlinks to related groups and experiments, and downloadable raw data from instruments.

For group cards, information on concentrations and metadata is aggregated from the samples included in the group and combined into tables. Additionally, the AMDB generates human-readable statistical charts for groups, where users can overview the data. These charts are Principal Component Analysis (PCA) 2D and 3D score plots, PCA 2D loading plots, Pie charts, Box plots, and Metadata analysis plots. Users can customize or download these charts, allowing for options such as selecting specific metabolites for analysis, adjusting data scaling, grouping types, including or excluding samples, and more (a complete list of functions can be found in the options).

**Figure 1:**
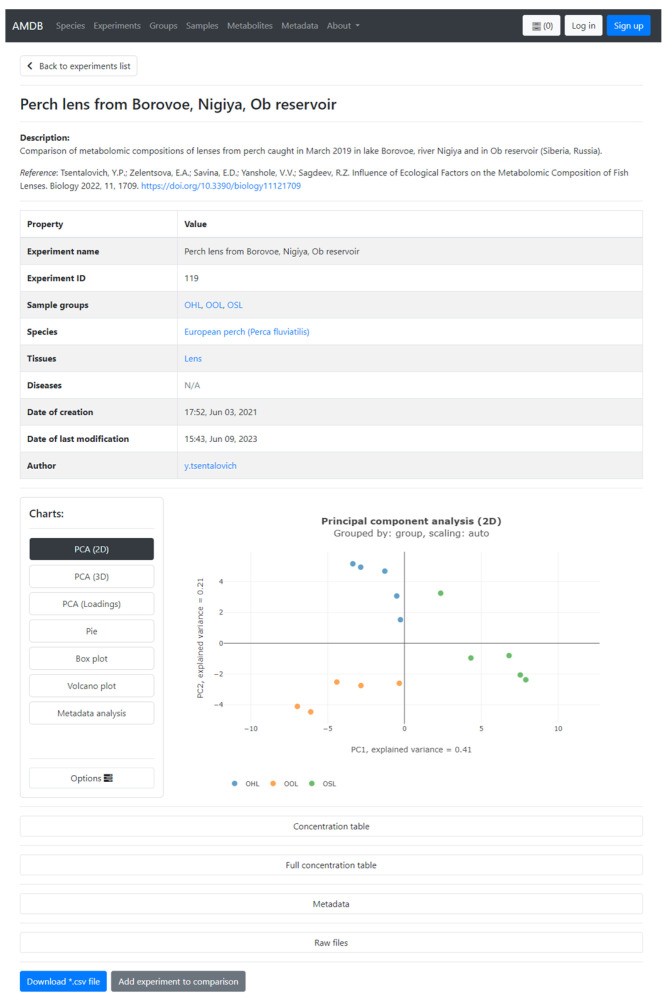
A screenshot of an experiment card. A card contains descriptions, metadata, dependencies, statistical charts, concentration data, metadata, and raw file tables (collapsed by default).

The AMDB hosts several ‘completed’ experiments, where two or more groups of samples have already been compared by our lab to determine differences in metabolomes resulting from various factors such as species, diseases, ecological factors, and more. Each experiment has a dedicated card (Figure 1), similar to the group card, which contains information gathered from the included groups. An additional table appears in which the average concentrations and standard deviations for the metabolites in the groups are calculated. In the statistical charts section, the Volcano plot becomes available.

To facilitate navigation and data management, samples, groups, and experiments are assigned unique ID numbers. Information from group and experiment cards, including data tables, statistical charts, and raw data, can be downloaded.

### 3.3. Metabolites

**Figure 2:**
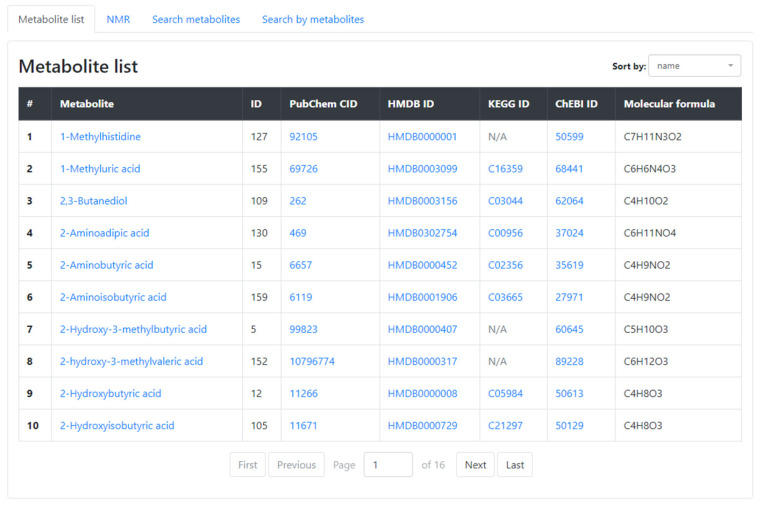
A screenshot from Metabolites section.

The **Metabolites** section of the AMDB (Figure 2) comprises a list of identified and quantified compounds. Each metabolite card provides a brief metabolite description, metabolite properties, links to external resources, and a search function for locating samples, groups, and experiments containing this particular metabolite. On the NMR page, users can access manually curated Nuclear Magnetic Resonance parameters for metabolites (Functional group, Multiplicity, Chemical shift, and Number of protons).

### 3.4. Comparing Groups from Different Experiments

**Figure 3:**
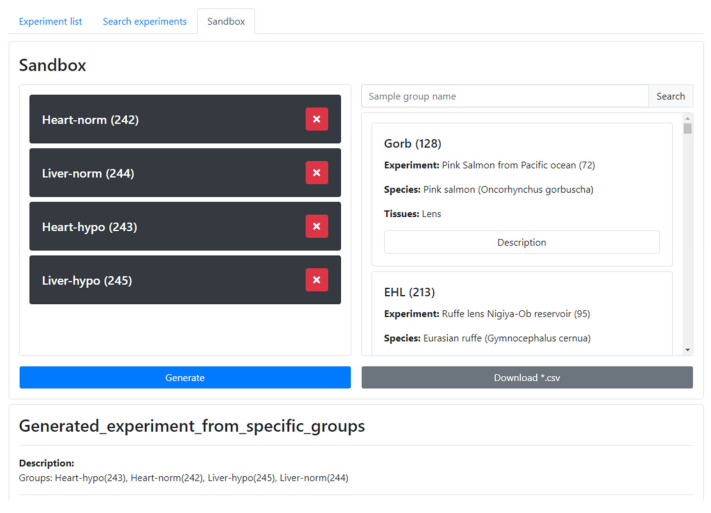
A screenshot from the **Sandbox**. Groups can be searched on the right part of the Sandbox and then selected groups can be grabbed and moved to the left part. The new experiment can then be generated.

To facilitate the comparison of groups from different experiments, the AMDB provides a convenient method called the **Cart**. To effectively use this feature, one can follow the steps below:

- Search for a group of interest and access its card.
- Scroll down to the bottom of the card, where you will find the ‘*Add group to comparison*’ button, and click it to add the group to the Cart.
- Repeat the process for searching and selecting additional groups to be compared.
- The Cart will display the list of selected groups, allowing one to review and make further modifications in selection if needed.
- To proceed with the comparison, click the ‘Compare’ button. This action will transfer the selected groups into the **Sandbox** (Figure 3).

Alternatively, groups can be added directly within the **Sandbox**. Search for groups using their names or IDs, and then add the groups of interest for comparison. Based on the selection, the system will consolidate the relevant information from the groups and combine it into a ‘new’ experiment with a standard card, with statistical charts, data tables, metadata tables, and raw data. The generated experiment and corresponding raw data can be downloaded. For completed experiments, all groups constituting the experiments can be added to the **Cart** by simply clicking the ‘*Add experiment to comparison*’ button at the bottom of the experiment card. By utilizing the **Cart** and **Sandbox** features, researchers can easily compare multiple groups of interest, enabling comprehensive analysis across different groups or experiments within the AMDB.

Similarly, the species comparison page in the AMDB consolidates all the information related to a specific tissue in one or multiple species of interest. This page generates a comprehensive summary similar to an experiment card.

## 4. Uploading Your Data and Comparing It with the Data Already Present in the AMDB

The AMDB offers registered Users the ability to upload their own quantitative metabolomic data and compare it with the data already available in the AMDB. There are two options for this: the first option is for a quick comparison of the User’s own data without publishing it in the AMDB, and the second option is for designing a more suitable data and metadata structure to conform to the AMDB. Registered Users can access the **Data Manager** section, where they can create and manage their own experiments.

### 4.1. Quick Comparison of User Data without Publication in the AMDB

To quickly compare the User’s own data with the existing data in the AMDB, one can perform the following steps:

- Download a simplified Excel template from the **Data Manager** section. This template contains only the required fields that need to be filled (groups, samples, and metabolites).
- Follow the instructions provided in the template. Fill in the rows with the metabolite names and their concentrations (in nmol/g) in the template. Empty and N/A values indicate difficulties in quantification (e.g., overlapping peaks) or omitted values. Zero values indicate that the value is below the LOQ of the instrument.
- Upload the completed template file, and a standard experiment card will be generated. The created experiment is private, and visible only to the User. The groups created by the User are now available for comparison with existing groups in the AMDB groups.

### 4.2. Uploading and Publishing User Data in the AMDB

**Figure 4:**
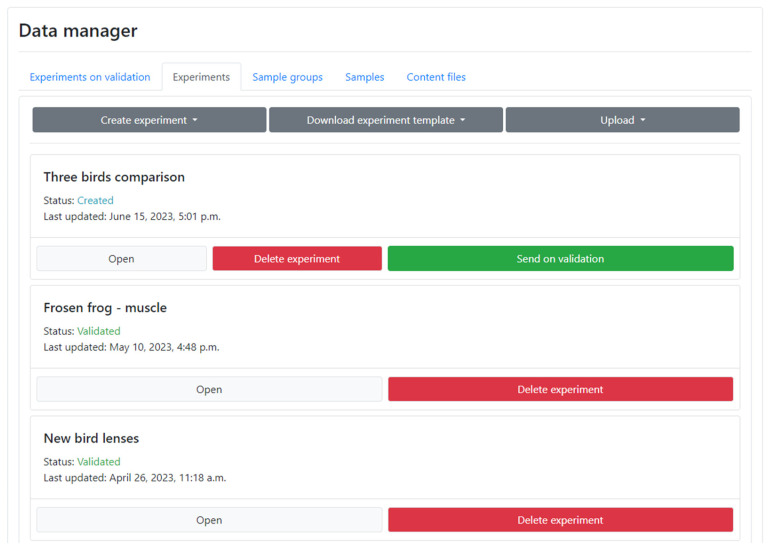
A screenshot from the **Data Manager** section. This section facilitates the uploading of User’s own data and its management within the AMDB.

To design a more appropriate structure for the data under submission, an extended template is available. This template includes additional fields for describing metadata, such as species, tissue, diseases, gender, origin, additional factors/attributes, and more. If everything is correct, the User can publish the experiment in the AMDB by submitting it for validation on the **Data Manager** page (Figure 4). If necessary, the User can update the uploaded experiment with a corrected version of the template; the corresponding button can be found at the bottom of the experiment card. Once the submitted experiment is validated, it will become publicly accessible in the database, and the corresponding samples, groups, and experiments will also be displayed in the User’s **Data Manager** section. The **Data Manager** also allows for the uploading of raw data (NMR or LC/GC-MS).

## 5. Database Application Examples

A portion of the data, published in the AMDB, has been previously applied in various scientific fields, including biology, chemistry, and medicine.

The first example is the application of the metabolomic approach in the study of a human eye disease, age-related nuclear cataract, characterized by opacification and coloration in the center of the eye lens. The lens is a hard-to-reach tissue, thus we compared cataractous lenses obtained as the post-operational material with control lenses obtained post-mortem [27]. We discovered a colossal altering of concentrations of many metabolites (Experiment #276), but these alterations were attributed not solely to the cataract-related changes, but also to post-mortem processes. We then examined lenses and surrounding aqueous humor samples in order to establish the contribution of each process, and found several groups of metabolites—antioxidants, ultraviolet (UV) filters, and osmolytes—that are either synthesized or actively pumped into the lens by the lens epithelial cells [26]. Reduced concentrations of these metabolites in cataract lenses indicates that the development of age-related nuclear cataract may occur due to dysfunction of the lens epithelial cells. We tried to avoid post-mortem changes by using model animals, e.g., rats, to study the cataracts. A study of rat lenses (Experiment #163) revealed that the set of metabolites required to protect the lens and maintain homeostasis is completely different from that of humans since rats are nocturnal creatures and the protection of eye tissues from solar radiation is not as important for them as for humans [25]. It is believed, that namely the low-molecular-weight components of the lens defense system are involved in the formation of cataracts in humans [31]. On the contrary, the lens metabolomic composition of macaque is shown to be much more similar to the human lens (Experiment #273).

The observation of significant post-mortem changes in ocular tissues has developed into the application of metabolomics in thanatochemistry [32,33] (Experiments # 242, #244, #245, #277; metadata analysis by “time post-mortem” attribute). This involved determining the properties of post-mortem changes in tissue metabolomic profiles, such as the magnitude of changes, alteration rates, direction of change (decrease or increase), linearity of concentration change, and data scattering. Several metabolites (hypoxanthine, choline, creatine, betaine, glutamate, and glycine) and metabolite sets (creatine, choline, and betaine) were proposed as candidates that can be used to determine the post-mortem interval, a parameter crucial in forensic science.

Several examples concern the discovery of novel compounds or unusual properties of compounds. We have found high concentrations of ovothiol A (OSH)—a most powerful natural antioxidant, in the lenses of various fish species [28,34] (Experiments #129, #140). OSH, prior to our studies, had been found only in tissues of invertebrates, and it was reported [35], that the pathway responsible for the OSH synthesis had been lost during the evolutionary mutations in vertebrates. The observation of high concentrations of OSH in fish lenses leaves open the question of the mechanism of its origin. OSH was also found in other fish tissues [29], although at lower concentrations (Experiment #129). Consequently, these findings suggest the existence of a specific mechanism dedicated to maintaining high OSH concentrations in the lens. In relation to fish, OSH can indeed be termed a ‘lenticular antioxidant’.

The unusually high concentrations of the well-known coenzyme NADH have been found in the lenses of certain species of birds of prey (black kite, common buzzard) and waterfowl (Podicipedidae family), as well as an extremely low [NAD^+^]/[NADH] ratio [36]. NADH has high absorption in the UV-A range with a maximum of 340 nm. Photochemical measurements have shown that NADH has the properties of a UV filter, and these properties are as good as those of UV filters in the human lens. Thus, NADH makes birds’ vision sharper by reducing chromatic aberrations (Experiments #145, #255).

The following examples are related to biological and environmental applications for AMDB data. We observed seasonal variations in the lens metabolomic composition in two species of fish [34] (Experiment #140). These variations are presumably determined by the fish’s nutrition and the levels of dissolved in water oxygen (DO). The most season-affected metabolites are osmolytes and antioxidants, and the most affected metabolic pathway is the histidine pathway. We have also studied the influence of ecological factors on the metabolomic composition of lenses of three fish species [37] (Experiment #119). We found that increased water acidity due to CO_2_ emissions and reduced DO caused significant changes in the fish lenses’ metabolomic compositions, including amino acids, organic acids, and energy metabolites.

AMDB data were used to evaluate metabolic pathway alterations in animals under extreme conditions. The concentrations of metabolites were measured in tissues of worms (Experiment #269), frogs (Experiments #109, #250, and #251), and salamanders (Experiment #253) [38,39,40,41] in normoxia and under conditions of either hypoxia or freezing. We have shown that extreme hypoxia results in a metabolic switch to different pathways of glycolysis with the accumulation of lactate, alanine, and succinate while freezing causes the accumulation of cryoprotectants: glycerol in salamanders, glucose in worms, and glucose and glycerol in frogs. Significant changes have also been observed in other metabolic cycles, including cellular energy generation, the Krebs cycle, and amino acid metabolism.

Quantitative metabolomic data can also be used in evolutionary science. We have shown that data from the AMDB (Experiment #145) can aid in the reconstruction of vertebrate phylogeny. The application of hierarchical clustering analysis (HCA) to the metabolomics data yielded dendrogram trees that match, although not perfectly, the genomics- and transcriptomics-based trees [30]. The conspecific samples in the HCA trees are placed near each other in a single cluster, regardless of the place and date of sampling. The tree structure of HCA confirms the key role of genomics in the formation of the lens metabolome but also indicates the influence of the species’ lifestyle. The combination of ’classical’ genomics-based phylogenies and younger metabolomics-based phylogenies has the potential to solve emerging problems in phylogeny and create a more robust tree of life.

## 6. Database Implementation

The AMDB website was developed using *Django v3.1.5*, an open-source *Python* web framework. *Python v3.9* was also utilized for developing the core functionalities of the AMDB. Data analysis within the AMDB is performed using specialized *Python* libraries, including *NumPy v1.20.1*, *SciPy v1.6.2*, and *Sklearn (Scikit-learn) v0.24.1*, which provide a range of tools for efficient data manipulation and statistical analysis. In terms of data storage and management, the AMDB utilizes *PostgreSQL v16.0* as a relational database management system. *PostgreSQL* enables effective handling of large data volumes, ensures data integrity, and supports complex queries, making it suitable for the requirements of the AMDB.

For the frontend design and user interface, we employed the *Bootstrap v4.6.2* toolkit (https://getbootstrap.com/, accessed on 7 October 2023), which provides a collection of responsive components and CSS styles, facilitating the development of a visually appealing and user-friendly website interface. Data visualization in the AMDB is achieved using *Plotly v2.16.1* (https://plotly.com/javascript/, accessed on 7 October 2023), a powerful library that enables interactive and customizable data visualizations. *Plotly* supports a wide range of charts and graphs, enhancing the presentation and exploration of data within the AMDB platform.

## 7. Conclusions, Limitations and Future Plans

The AMDB contains unique information on metabolite concentrations in animal tissues, gathered in one place. None of the existing databases allow for convenient searching and browsing of metabolite concentrations in various animal tissues. The second, but no less important function of the AMDB is to serve as a platform for the metabolomics community providing convenient deposition and exchange of quantitative metabolomics data. The AMDB is constantly growing, and its interface is developing. As mentioned above, the AMDB now primarily covers the metabolite content of the eye lens and blood of vertebrates, mainly wild species from Siberia, Russia and laboratory rodents. However, data on other tissues (muscle, heart, liver, brain, and more) are also present. Although the eye lens is a very convenient tissue for sampling and reflects in general the metabolome of a species, other more common tissues are planned to be added to the database by our group. Another planned extensive branch of the AMDB development is the inventory of laboratory animals widely used in experiments, such as common mouse strains (C57BL, BALB, DBA), rats (Sprague Dawley, Long-Evans, Fischer 344), guinea pigs, zebrafish, etc. The contribution of other scientific groups to this branch is highly appreciated. The metabolite levels in tissues can be used as a baseline in comparative experiments.

The AMDB web interface is moving towards simplifying data uploading and the publication of newly mined scientific discoveries. The AMDB is also moving towards FAIR (Findability, Accessibility, Interoperability, and Reusability) compliance [42] and incorporation into common pipelines and community-driven techniques and formats such as CSV, JSON, XML, featureXML and nmrML, to allow better interoperability [18,43,44,45,46,47,48,49].

The AMDB team is eager to encourage scientific groups around the world to deposit their quantitative metabolomic data for animal tissues. We lay our hopes on this contribution to the AMDB database and platform, which will lead to more comprehensive data coverage, making the AMDB a widely used tool to meet the emerging needs of the metabolomics community.

## Data Availability

The data are deposited at https://amdb.online.
